# Human Cytomegalovirus Inhibits Autophagy of Renal Tubular Epithelial Cells and Promotes Cellular Enlargement

**DOI:** 10.3389/fcimb.2020.00474

**Published:** 2020-09-15

**Authors:** Ana C. López Giuliani, Eva Hernández, María J. Tohmé, Clémence Taisne, Julieta S. Roldán, Clara García Samartino, Marion Lussignol, Patrice Codogno, María I. Colombo, Audrey Esclatine, Laura R. Delgui

**Affiliations:** ^1^Facultad de Ciencias Médicas, IHEM, Universidad Nacional de Cuyo, CONICET, Mendoza, Argentina; ^2^Facultad de Ciencias Exactas y Naturales, Universidad Nacional de Cuyo, Mendoza, Argentina; ^3^Université Paris-Saclay, CEA, CNRS, Institute for Integrative Biology of the Cell (I2BC), Gif-sur-Yvette, France; ^4^Facultad de Farmacia y Bioquímica, Universidad Juan Agustín Maza, Mendoza, Argentina; ^5^Instituto de Investigaciones Biotecnológicas (IIBIO), Universidad de San Martín, Buenos Aires, Argentina; ^6^Facultad de Ciencias Médicas, Universidad Nacional de Cuyo, Mendoza, Argentina; ^7^Institut Necker-Enfants Malades (INEM), INSERM U1151-CNRS UMR 8253, Paris, France; ^8^The Université Paris Descartes, Sorbonne Paris Cité, Paris, France

**Keywords:** human cytomegalovirus, renal cells, autophagy, primary cilium, cellular size, cytomegaly, cytopathic effect, polarization

## Abstract

Human Cytomegalovirus (HCMV) is a frequent opportunistic pathogen in immunosuppressed patients, which can be involved in kidney allograft dysfunction and rejection. In order to study the pathophysiology of HCMV renal diseases, we concentrated on the impact of HCMV infection on human renal tubular epithelial HK-2 cells. Our aim was to develop a model of infection of HK-2 cells by using the viral strain TB40/E, that contains the extended cell tropism of clinical isolates and the efficient viral multiplication in cell culture of laboratory-adapted strains. We observed that HK-2 cells can be infected by HCMV and expressed viral antigens, but they do not produce extracellular viral particles. We then studied the interplay of HCMV with ciliogenesis and autophagy. Primary cilium (PC) is a stress sensor important to maintain renal tissue homeostasis that projects from the apical side into the lumen of tubule cells. PC formation and length were not modified by HCMV infection. Autophagy, another stress response process critically required for normal kidney functions, was inhibited by HCMV in HK-2 cells with a reduction in the autophagic flux. HCMV classically induces an enlargement of infected cells *in vivo* and *in vitro*, and we observed that HCMV infection led to an enlargement of the HK-2 cell volume. Our results constitute therefore an excellent starting point to further explore the role of these mechanisms in renal cells dysfunction.

## Introduction

HCMV is a ubiquitous double-stranded DNA virus belonging to the *Herpesviridae* family. Its name is derived from the observed enlargement of the infected cell (cytomegaly) (Mocarski et al., 2001). Like other viruses of this family, after primary infection it establishes a latent infection within the host, with a possibility of reactivation. Its prevalence is high in the human population (almost 100% of the adults in developing countries have antibodies against HCMV). In healthful individuals, primary infection is oftentimes asymptomatic and then a persistent infection developed, managed by the host immune system (Boeckh, 2011). Even though, HCMV is a major cause of morbidity and mortality in individuals with impaired immune systems. Transplant patients receiving immunosuppressive chemotherapy are at an increased risk of active HCMV infection and disease. As a result, despite antiviral preventive strategies, HCMV infection is an important complication in individuals receiving solid organ transplants, including kidney recipients (Carney, 2013). Kidney transplant is the most common organ transplant surgeries performed nowadays and HCMV infection is a regular complication, which usually appears during the first year after renal transplantation, notably when a seronegative allograft recipient receives a kidney from a HCMV seropositive donor (Morgantetti et al., 2019). The effect of HCMV on the destiny of solid organ transplantation is significant, not only causing grave and potentially lethal illness, but also promoting allograft rejection, additional opportunistic infections, post-transplant lymphoproliferative disorders, vascular disease, and the entire patient and allograft survival (Razonable and Humar, 2013).

HCMV infection has direct and indirect effects on the patient and the graft (López-Oliva et al., 2017). The direct effects of HCMV are associated with viral replication and HCMV infection/disease. HCMV is well-known to cause tubulointerstitial nephritis with cytopathic changes in the tubular epithelial cells and endothelial cells of peritubular capillaries (Rane et al., 2012). HCMV glomerulopathy and associated kidney dysfunction may occur despite prophylactic therapy. The indirect effects have been associated with increased morbidity (opportunistic infections such as *Pneumocystis jirovecii* pneumonia and invasive aspergillosis), graft loss and long-term mortality.

We have been working for several years on the characterization of the interplay between HCMV and autophagy (Chaumorcel et al., 2008, 2012; Mouna et al., 2016; Taisne et al., 2019). Autophagy is a conserved cellular pathway involved in cellular homeostasis maintenance and in cell survival in response to stress. It constitutes a catabolic process through the lysosomal pathway, leading to the degradation of long-lived proteins and damaged organelles. In this article, we focused on macroautophagy (hereafter referred as autophagy) during which, a phagophore forms and sequesters cytoplasmic constituents, including organelles. Then, the edges of the phagophore fuse to form the autophagosome, a double membrane vesicle. The autophagosome undergoes fusion with a lysosome to form an autolysosome, where degradation occurs. This whole process is tightly regulated (Esclatine et al., 2009). Briefly, under starvation, a classical inducer of autophagy, a protein complex localized at the forming phagophore is responsible for the production of phosphatidylinositol 3-phosphate (PI3P), necessary for vesicle growth. This complex is mainly formed by ATG14, BECLIN 1 and VPS34 proteins. The phagophore then elongates to fuse from both extremities forming the autophagosome, which matures along the endocytic pathway, finally fusing with a lysosome to generate a degradative autolysosome. The cytoprotective role of autophagy has been well-documented in different non-infectious nephropathies, although the precise mechanism of autophagy on kidney fibrosis remains elusive (Huber et al., 2012; Kaushal and Shah, 2016). Various harmful stimuli such as renal ischemia-reperfusion (I/R), sepsis, or exposure to nephrotoxins lead to nutrient depletion and oxidative stress–dependent activation of autophagy (Sureshbabu et al., 2015). Accumulating evidence in various rodent models, such as I/R-induced, sepsis/endotoxemia-induced, and nephrotoxin-induced acute kidney injury, strongly suggest that autophagy generally protects the kidney from injury, though contrasting findings have also been reported (Choi, 2019). Interestingly, it has been demonstrated that autophagy can be induced in epithelial cells by primary cilium (PC) activation triggered by fluid flow (Orhon et al., 2016b; Zemirli et al., 2019). This activating pathway notably regulates epithelial cell size. We and others have previously shown that HCMV infection of human fibroblasts modulates autophagy and we recently demonstrated that HCMV can block autophagosome maturation in order to subvert the autophagic machinery to its own profit (Mouna et al., 2016; Taisne et al., 2019). However, it has been reported that modulation of autophagy by viruses can be cell-type dependent.

We thus explored whether HCMV can modulate autophagy in renal epithelial cells and whether this regulation could contribute to renal physiopathology in the context of HCMV infection during kidney transplantation. We have specifically chosen to work with HK-2 cells, an immortalized human cell line, because HCMV only infects human cells and HK-2 cells retain functional characteristics of proximal tubular epithelium (Ryan et al., 1994). We observed that HK-2 cells are permissive to HCMV, although they do not produce extracellular viral particles. In this report, we have approached the impact of HCMV on ciliogenesis, autophagy, and cell volume of HK-2 cells.

## Materials and Methods

### Cells and Viruses

MRC-5 primary human embryonic lung fibroblasts were purchased from Biomérieux and employed between passages 23 and 28 post-isolation. MRC-5 cells were grown in minimum essential medium (MEM) (Gibco, 21090-055) supplemented with 10% fetal calf serum (FCS) (Gibco, 10270-106), penicillin G (100 U/ml), streptomycin sulfate (100 μg/ml), l-glutamine (1%), and non-essential amino acids (1%). Immortalized human epithelial kidney HK-2 cells, provided by Fabiola Terzi (INEM, Paris), were maintained in DMEM supplemented with 10% FCS, penicillin G (100 U/ml), streptomycin sulfate (100 μg/ml), EGF (Sigma, E9644), hydrocortisone (Sigma, H0135), T3 (Sigma, T5516) and ITS (Sigma, I3146). Human epithelial cervix cancer (HeLa, ATCC CCL-2) cell line was cultured in Dulbecco's modified Eagle's medium (DMEM) (ThermoFisher Scientific, 12-800058) containing 10% FCS (Gibco, 10270-106). The culture media was supplemented with penicillin/streptomycin (10,000 U/ml; ThermoFisher Scientific, 15140122). The three cell lines were grown at 37°C and under a 5% CO_2_ atmosphere. HCMV AD169 strain was purchased from ATCC and propagated in MRC-5 cells as described (Esclatine et al., 2001). HCMV TB40/E strain was gently provided by Christian Sinzger (Institute for Virology, Ulm University Medical Center) and was propagated in HUVEC cells and them amplified in MRC-5 cells (Sinzger et al., 1999). Experiments were performed in the context of Biosafety Level 2 laboratory.

### Autophagy Modulation

All autophagic parameters and treatments were performed as described (Mouna et al., 2016). Briefly, starvation-induced autophagy was carried out by culturing the cells in Earle's Balanced Salt Solution (EBSS) (Gibco, 24010-045) for 4 h before fixation or lysis. Where indicated, cells were treated with Spautin-1 (20 μM, Sigma-Aldrich, M9281). To inhibit autophagic flux, cells were treated for the indicated times with chloroquine (CQ) (50 μM, Sigma-Aldrich, C6628).

### Antibodies

To identify HCMV-infected cells, we used mouse monoclonal antibodies against the viral proteins IE1 and IE2 (clone E13; Biomérieux, 11–003). Additional primary antibodies used included anti-ZO-1 (Zonula Occludens) (provided by Isabelle Beau, Inserm UMR-S 1185, Le Kremlin Bicêtre, France), anti-γ-tubulin (Santa Cruz Biotechnology, sc-10732), anti-acetylated tubulin (Sigma-Aldrich, T7451), anti-LC3 (MBL-PM036), anti-LC3B (Sigma L7543, used for immunoblot analysis), anti-β-actin (Merck Millipore MAB1501 clone C4) and 4′,6-diamidino-2-phenylindole (DAPI) (Invitrogen, D1306). Secondary antibodies included Alexa Fluor 555 goat anti-mouse and anti-rabbit (Life technology, A21424 – A21428), Alexa Fluor 488 goat anti-rabbit and anti-mouse (Jacskon ImmunoResearch, 111-545-003 and 115-545-003), Alexa Fluor 647 donkey anti-mouse (life technology, A32787). Horseradish peroxidase (HRP)-labeled goat anti-mouse and anti-rabbit secondary antibodies were purchased from Jackson ImmunoResearch Laboratories (115–035–003, 111–035–003).

### HCMV Infection and Titration

HCMV suspended in serum-free medium, or serum-free medium alone (mock-infected) was adsorbed onto cells for 1 h at 37°C at a multiplicity of infection (MOI) of 1. After removing the inoculum, the cells were cultured in medium containing 10% FCS and processed for the different assays at the indicated times post-infection (p.i.). For viral production analysis, viruses were adsorbed onto cells for 1 h at 37°C. After removal of the inoculum, infection was synchronized using citrate buffer (40 mM citric acid, 10 mM KCl, 135 mM NaCl, pH = 3) to inactivate non-internalized viruses. At the indicated times, viruses were collected from cell-free supernatants and quantified as previously described (Mouna et al., 2016).

### Immunoblot Analysis

HK-2 and MRC-5 cells were lysed in 65 mM Tris, pH 6.8, 4% SDS, 1.5% β-mercaptoethanol, and incubated at 100°C for 5 min. Proteins extracts were resolved on SDS-PAGE gels (12.5%) and transferred onto a polyvinylidene difluoride membrane (Perkin Elmer). Membranes were blocked and probed with primary antibodies overnight. HRP-labeled antibodies were used as a secondary antibody, revealed using the ECL detection system following the manufacturer's instructions (Immobilon, Millipore) and anti-actin was used to verify equal loadings. Quantification of protein levels was performed using Photoshop CS5 software (Adobe systems).

### Immunofluorescence Analysis

Cell monolayers were washed with phosphate buffered saline (PBS), fixed with 4% paraformaldehyde in PBS, permeabilized using 0.05% Saponin in PBS, incubated for 1 h in PBS supplemented with 5% of goat serum for blocking, and then with the appropriate primary antibodies. The cells were washed 3 times in PBS Tween 20 0.1%, and then incubated with appropriate secondary antibodies. Coverslips were mounted in Glycergel (Dako, C0563) and examined using a Zeiss Axiovert 200 M epifluorescence microscope (Zeiss instruments) or a Zeiss ApoTome 2 microscope piloted with Zen software 2012 or with a confocal Leica TCS SP5 (LAS AF version 2.6.0; laser wavelengths 488 nm, 561 nm and 633 nm) (SFR Necker, Cell Imaging Platform). Images were resized, organized, and labeled using Photoshop software.

### Quantification and Statistical Analysis

The number of endogenous LC3-positive dots per cell was established using ImageJ software. Images were converted into 8-bit and brightness/contrast adjusted to increase the accuracy of the count. A thresholding of images was performed to detect only LC3 vesicles. Finally, the “analyse particles” plug-in was used to quantify the number of LC3-positive dots per cell. For each condition, at least 25 cells were analyzed in three independent experiments.

The mean cell volume was calculated employing Imaris software 1.47n (NIH). We used over-saturated LC3 stained images to observe the complete cell and a Zeiss ApoTome 2 microscope to acquire the z-stack images of the cells in different visual fields of the μ-slide. Data came out from three independent experiments with 25 counted cells in 5 different visual fields for each experiment. 3D reconstruction images were modeled using Imaris Software and number of ciliated cells and the length of cilia were quantified using the same software. For PC lengths distribution analysis, we established two intervals of lengths (0–1.15 and 1.16–12 μm), and quantified the number of PCs, out of 150 total structures, in each interval.

Data are expressed as means ± standard error (SE) and were analyzed with Infostat program by using Student's *t*-test or two-tailed analysis of variance (ANOVA) with Tukey's test contrast. *P* < 0.05 were considered statistically significant. Experiments were performed a minimum of three times.

### Flow Cytometry Analysis

HeLa cells were treated with CQ 50 μM, or left in control condition, for 4 h before direct analysis of the cells. These assays were performed by using the FACSARIA-III (BD Biosciences) equipped with 488 nm and 633 nm lasers from the Flow Cytometry Facility of the “Facultad de Ciencias Médicas, Universidad Nacional de Cuyo” (Argentina). Data was analyzed using FlowJo 7.6 software.

## Results

### HCMV Strain TB40/E Infects HK-2 Renal Tubular Epithelial Cells

**Figure 1:**
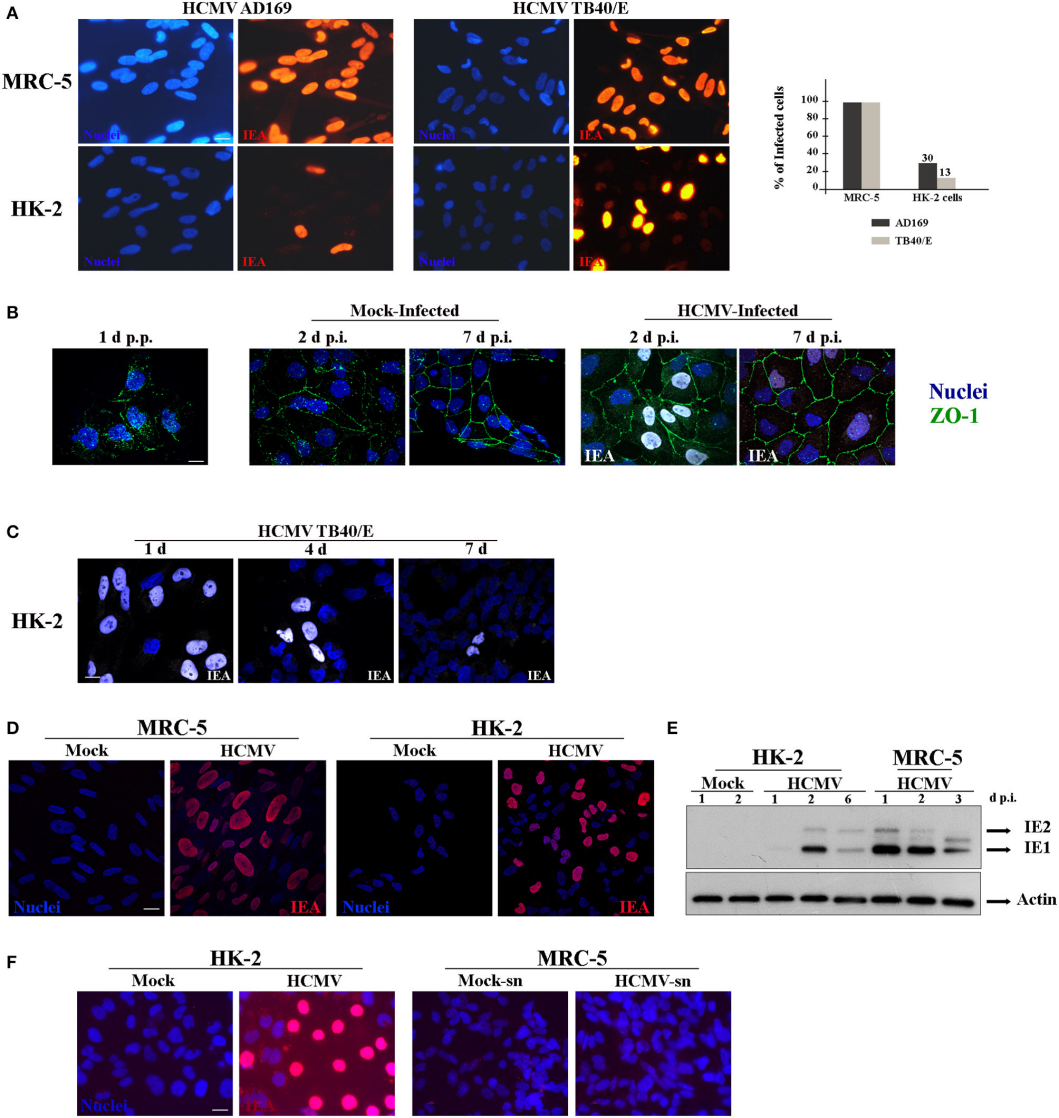
Characterization of infection of HK-2cells by HCMV. **(A)** Representative images of MRC-5 and HK-2 cells infected with HCMV at MOI 1 during 48 h and immunostained for IEA (immediate-early viral antigens, in red). Two different strains, AD169 and TB40/E, were tested. Nuclei were subsequently stained with DAPI. **(B)** Representative images of HK-2 cells infected with HCMV or mock-infected for 2 or 7 days, and immunostained for IEA (in white) and ZO-1 (in green) to visualize cell polarization. Lack of polarization of HK-2 cells 24 h post plating (p.p.) is depicted (left panel). **(C)** Representative images of HK-2 cells infected with HCMV at MOI 1 at different times p.p. (1 to 7 days) and immunostained for IEA (white) and the nucleus. **(D)** Confocal images of MRC-5 and HK-2 cells infected with HCMV for 24 h and immunostained for IEA (red) and the nucleus. **(E)** Immunoblot analysis of IE1 and IE2 after HCMV infection at MOI 1 of MRC-5 or HK-2 cell lines at 1, 2, 3, or 6 days post infection (d p.i.). Actin was used as a loading control. **(F)** Representative images of HK-2 cells infected with HCMV for 7 days and immunostained for IEA (left panels). The non-diluted supernatants from infected (HCMV-sn) or from mock-infected HK-2 cells were tested on MRC5 cells in order to detect viral production by IEA expression (right panels). Scale bars = 10 μm. Three independent experiments were performed and statistical analysis was achieved as explained in the materials and methods section.

Since our studies were focused in elucidating HCMV-induced renal pathophysiology, we used the established HK-2 cell line of human immortalized renal tubular epithelial cells (Ryan et al., 1994). To the best of our knowledge, only one study has previously employed HK-2 cells to explore HCMV renal pathogenic mechanisms (Shimamura et al., 2010). Using HCMV TR strain, they demonstrated that infection of HK-2 cells induced TGF-β1 production, a fibrogenic cytokine, which might contribute to the renal fibrosis observed in the context of infection of transplanted kidney. Nevertheless, the mechanisms involved in renal HCMV disease remain largely undefined. However, in this study, they observed that TR virions were not released in the supernatant and remained cell-associated, and the maximum titer obtained was much less than in human fibroblasts (Shimamura et al., 2010). Then, we decided to use an alternative HCMV clinical strain, which exhibits a broad cell tropism and is widely used for infection of endothelial and monocyte-derived cells, named TB40/E (Sinzger et al., 1999, 2008). We thus first investigated the permissivity of HK-2 cells to the highly passaged fibroblast-adapted laboratory strain AD169 and the strain TB40/E. Primary human embryonic lung fibroblasts (MRC-5 cells), a well-established cellular system for HCMV studies, and HK-2 cells were infected in parallel with HCMV at a multiplicity of infection (MOI) of 1. Cells were fixed 48 h post-infection (h p.i.), immunostained for immediate-early viral antigens (IEA) in order to visualize infected cells, and analyzed by epifluorescence microscopy. A large proportion of the MRC-5 cells were positive for IEA with both viral strains (Figure 1A, upper panels). In HK-2 cells, 13 and 30% of the cells were positive for IEA when infected with AD169 or TB40/E strain, respectively (Figure 1A, lower panels), showing that TB40/E viruses were more efficient to enter HK-2 cells. We thus decided to use TB40/E strain for the following experiments. The urinary tract (kidney, urinary bladder) is formed by a single layer of polarized epithelial cells. HK-2 cells start polarizing as soon as they reach confluency over the substratum, thus mimicking the morphology of the urinary track epithelia. We decided to investigate both the impact of HCMV infection on polarization and the impact of polarization on the permissiveness of HK-2 cells to HCMV. In order to visualize HK-2 polarization, we employed antibodies against zonula occludens protein-1 (ZO-1), a tight junction protein (Shin et al., 2006). We observed that HK-2 cells were completely polarized after 7 days but the polarization process was not affected by HCMV-infection (Figure 1B). Thus, to explore whether the virus was able to enter the cells once polarized, HK-2 cells were seeded in sub confluency and infected with HCMV at an MOI of 1 on day 1, 4, and 7 p.p. and we left the infection to proceed for 48 h. Afterwards, the cells were fixed and immunostained for IEA. As shown in Figure 1C, a greater rate of infection was obtained when infection was performed on non-polarized cells (1 day p.p.), while a decreased infectivity was observed when the infection was performed on a polarized monolayer of cells at 7 days p.p. Then, from now on, the infections were always done 1 day p.p. Next, to analyze the intracellular distribution phenotype of IEA expression in detail, we used laser scanning confocal microscopy (LSCM). MRC-5 and HK-2 cells were infected in parallel with HCMV and fixed at 24 h p.i., and immunostained for IEA. As shown, a similar nuclear distribution was observed for both cell types (Figure 1D). We also comparatively analyzed the kinetics of IEA expression level in MRC-5 and HK-2 cells. The cells were mock- or HCMV-infected in parallel and at 1, 2, 3, and up to 6 days p.i., the cells were lysed and the intracellular levels of IEA were analyzed by Western blot. In MRC-5 cells, we observed expression of IEA (IE1 & IE2) from 1 day p.i. with a decreased accumulation at 3 days p.i., probably due to protein degradation (Figure 1E). At 6 days p.i., cells were totally lysed by HCMV. In HK-2 cells, we observed accumulation of both IE1 and IE2 at 2 days p.i. and a decreased expression at 6 days p.i., which can be interpreted as a delay in the infectious cycle in HK-2 cells (Figure 1E). However, we could not detect expression of pp28, a late tegument viral protein, classically expressed after viral replication, suggesting that either pp28 is expressed at a low level or the viral cycle is incomplete (data not shown). Finally, in order to decipher whether HCMV infection of these cells was productive (i.e., generation of viral particles), we analyzed a 1-week long infection of HK-2 cells. Supernatants from HK-2-infected cells were titrated using MRC-5 cells. Whereas, HK-2 cells were infected, as shown with IEA staining (Figure 1F, left panels), we did not observe any MRC-5 cells positive for IEA, suggesting that there was no HCMV production in HK-2 cells after one-week (Figure 1F, right panels). Taken together, our data demonstrated that HCMV strain TB40/E was able to enter HK-2 cells and to express IEAs, although in a delayed kinetics compared to MRC-5 cells, but it is incapable of propagating in these cells.

### HCMV Infection Does Not Affect Ciliogenesis of Renal Tubular Epithelial Cells

**Figure 2:**
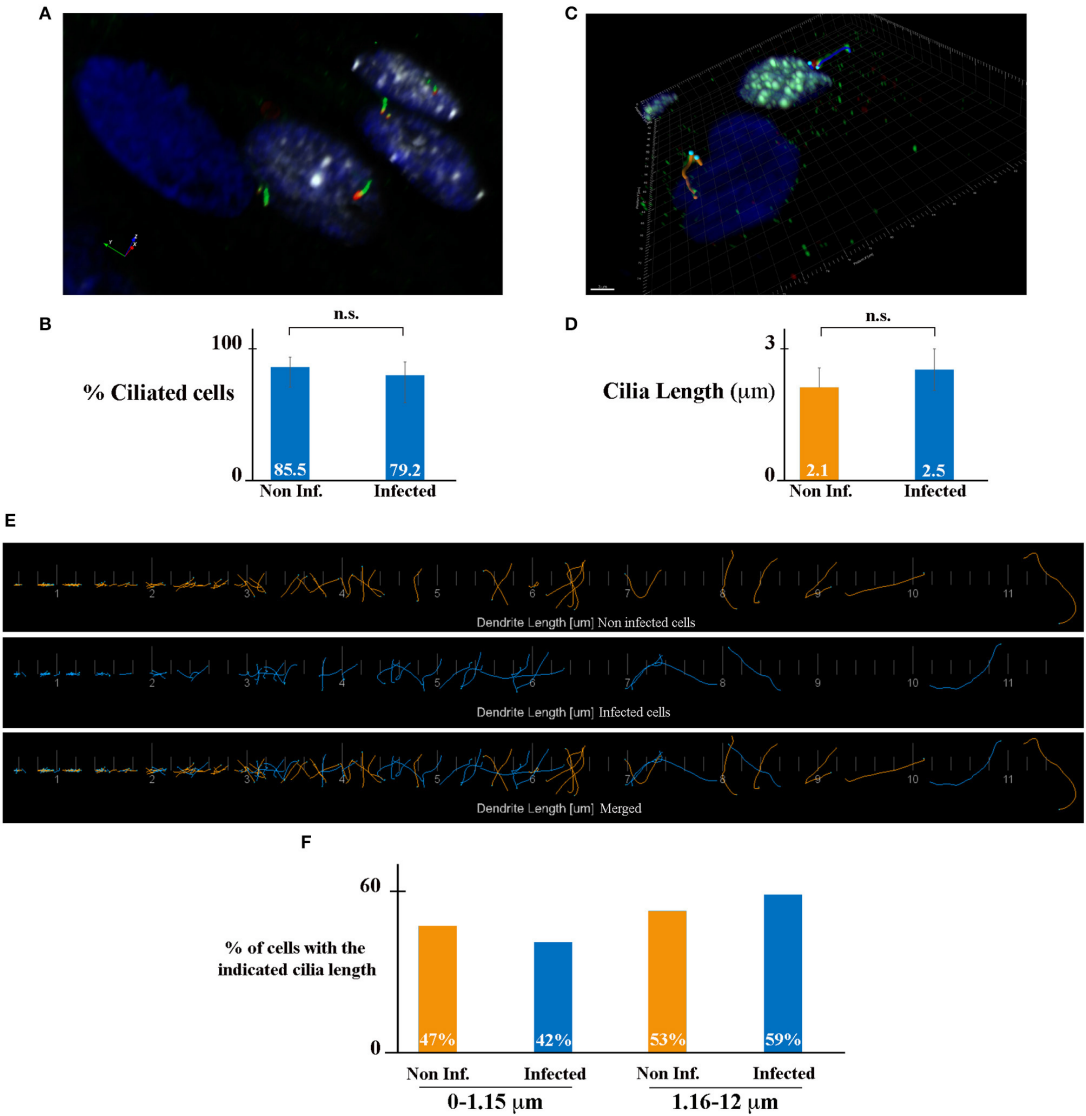
HCMV does not affect ciliogenesis of HK-2 cells. **(A)** SIM image of HCMV-infected polarized HK-2 cells immunostained for IEA (white), acetylated tubulin (PC axoneme marker, green), and γ-tubulin (PC basal body marker, red). Nuclei were subsequently stained with DAPI. **(B)** Percentage of ciliated cells of non-infected and infected HK-2 cells. **(C)** Representative 3D image of ciliated non-infected (blue nucleus) and HCMV-infected (white nucleus) HK-2 cells. The length of PC of each cell was visualized with acetylated tubulin and γ-tubulin and measured with Imaris Software. Scale bar represents 3 μm. **(D)** Quantification of cilia length in non-infected and infected HK-2 cells. PC length of 150 cells per condition was measured. n.s, non-significant (Student's *t*-test). **(E)** Visualization of every PC from non-infected (orange) or infected (blue) cells (separately and merged) listed in increasing order. **(F)** PCs were separated based on their length in two categories (0–1.15 μm) and (1.16–12 μm). Percentage of PC in these two categories in non-infected and infected cells. Three independent experiments were performed and statistical analysis was achieved as explained in the materials and methods section. n.s, non-significant (Student's *t*-test).

The primary cilium (PC), which is formed by a basal body and an axoneme, is a microtubule-based organelle at the surface of various cell types and is critical in maintaining tissue homeostasis (Malicki and Johnson, 2017). In the kidney, PC plays an important role in the maintenance of renal function and structure. All kidney tubular epithelial cells have a PC (one per cell). The PC protrudes from the apical surface of cells into the lumen of tubules, detects the fluid flow and composition, and maintains the architecture of tubule cells (Praetorius, 2015). It has been shown that the length of the PC is dynamically changed in cells as a result of various kidney diseases including acute kidney injury and chronic kidney disease (Park, 2018). We hypothesized that HCMV-infection may affect PC length in kidney epithelial cells, leading to an impact of its function and to viral-induced renal physiopathology. To address this question, we analyzed the presence and the length of PC in HCMV-infected HK-2 cells. We immunostained PC using antibodies against acetylated tubulin (axoneme marker, green) and γ-tubulin (basal body marker, red), and infected cells using antibodies against IEA (white) (Figure 2). We decided to let the infection proceed for 7 days in order to assure a confluent, polarized monolayer with maximum ciliogenesis. The samples were analyzed by structured illumination microscopy (SIM). As shown in Figure 2A, HCMV-infected cells presented analogous PCs to neighboring non-infected cells. We quantified the number of cells presenting a PC, and we observed a similar percentage of ciliated cells in non-infected (blue nucleus) and HCMV-infected cells (white nucleus). Indeed, in non-infected and in HCMV-infected cells 85.5 and 79.2% of cells presented a PC, respectively (Figure 2B). Next, to evaluate the impact of infection on the PC length, IF images were used to highlight the PC structures in non- and HCMV-infected cells (Figure 2C). The mean PC length of non-infected cells, which was 2.1 μm, was not significantly different of the PC length of HCMV-infected cells (2.5 μm) (Figures 2D,E). In addition, the length distribution in both conditions was analyzed. Since the range of PC length was extremely extended (0.1–12 μm), it might be possible that a total mean value could miss significant differences between infected cells and non-infected cells. To go further, we decided to arbitrarily divide our data between “large PCs” (1.16–12 μm) and “small PCs” (0–1.15 mm). As observed in Figure 2F, a slight difference in the distribution was observed, although statistically non-significant. All these data taken together indicate that ciliogenesis of renal tubular epithelial cells was not affected by HCMV infection.

### HCMV Inhibits the Autophagy Pathway of Renal Cells

**Figure 3:**
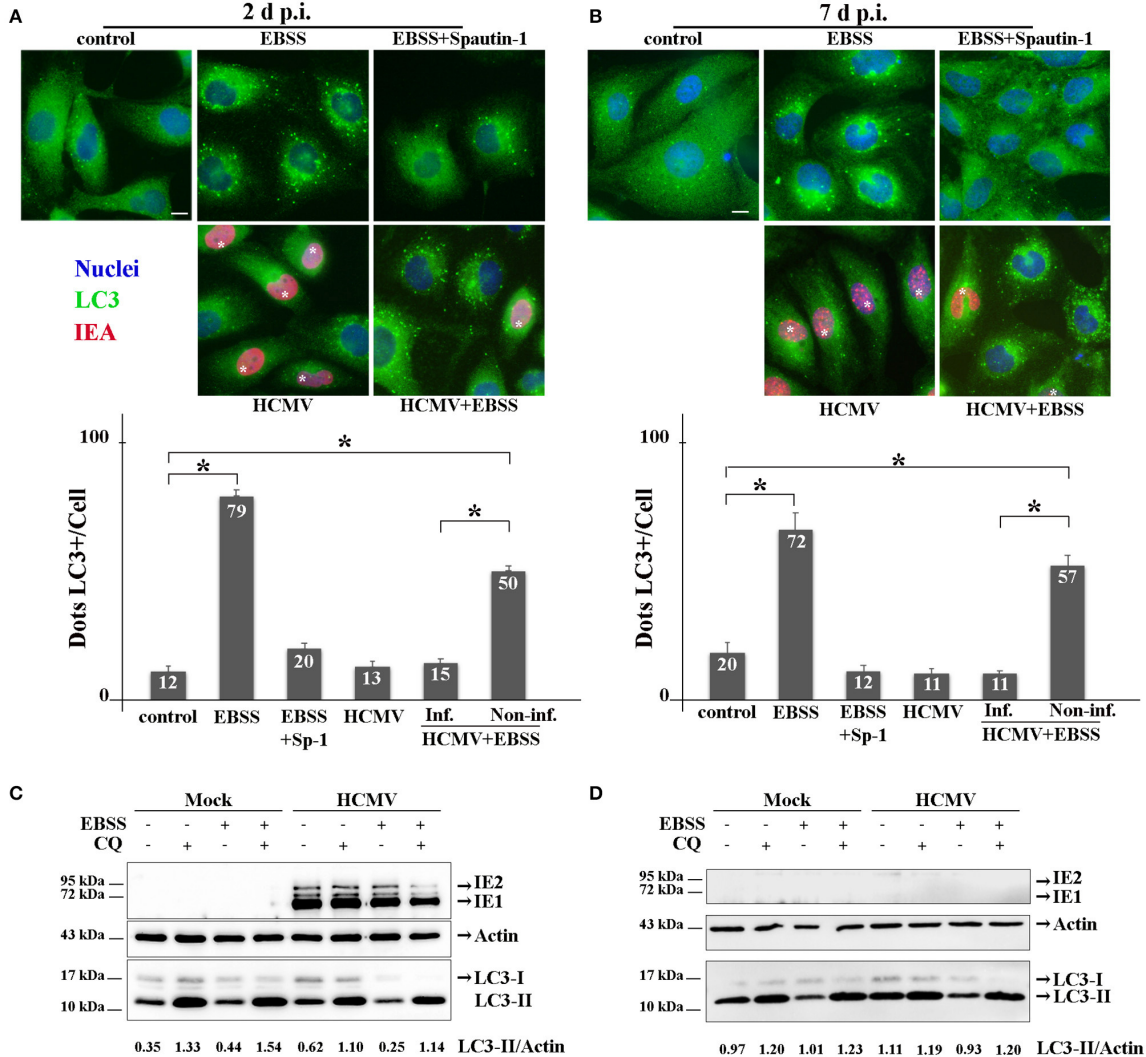
HCMV inhibits the autophagic process. **(A,B)** Representative images of HK-2 cells treated or not (control) with EBSS (to induce autophagy) or treated with EBSS + Spautin 1 (to block starvation-induced autophagy). HK-2 cells were also infected with HCMV at MOI 1 (and treated with EBSS for the 4 last h of infection or not). Cells were fixed after 2 days **(A)** or 7 days **(B)** and immunostained for LC3 (autophagosome marker, green) and IEA (viral protein, red). Nuclei were subsequently stained with DAPI. Scale bars represent 10 μm. Infected cells are marked with *. Quantification of LC3-positive dots per cell in each condition (lower panels). In HCMV + EBSS condition, cells expressing IEA (inf.) and neighboring non-infected cells (non-inf.) were analyzed separately. *p < 0.05 (ANOVA, with a Tukey contrast). **(C,D)** Immunoblot analysis of IEA and LC3-I and II in mock- or HCMV-infected HK-2 cells (2 and 7 days p.i.). Cells were treated when indicated for 4 h with EBSS and/or chloroquine (CQ). Actin was used as a loading control. A representative Western blot is depicted and LC3-II/Actin ratio is shown below the blot.

The interplay between HCMV and the autophagy pathway has been extensively studied by our group and others' [reviewed in Lussignol and Esclatine (2017)]. In fibroblasts, HCMV first stimulates autophagy quickly after its entry and subsequently blocks autophagosome maturation in order to form its final envelope (Mouna et al., 2016; Taisne et al., 2019). Because viruses modulate autophagy differently depending of the cell type, the impact of HCMV on autophagy was analyzed in HK-2 cells (Figure 3). Firstly, HK-2 cells were grown on coverslips and mock- or HCMV-infected for 48 h (non-polarized cells) or 7 days (polarized cells). Then, the cells were fixed and immunostained employing antibodies to LC3, a well-established autophagosome marker (Klionsky et al., 2016) (green) and to IEA (red) to visualize the autophagic structures and the infected cells, respectively. As controls, mock-infected cells were grown in EBSS, to induce autophagy by starvation, and treated or not with Spautin-1, used to counteract starvation-induced autophagy. Indeed, Spautin-1 constitutes a potent and specific inhibitor of autophagosomes biogenesis (Liu et al., 2011). The samples were analyzed by SIM and autophagic structures (LC3 positive dots) were quantified as described in the materials and methods section. We observed numerous LC3-positive dots when HK-2 cells were starved using EBSS, both in non-polarized and polarized cells, and Spautin-1 was able to block starvation-induced autophagosome formation. Then, HK-2 cells were HCMV-infected for 48 h (non-polarized cells) or 7 days (polarized cells). Although HCMV infection had almost no impact on basal autophagy, it was able to block starvation-induced autophagy in cells expressing IEA in non-polarized and polarized HK-2 cells (Figures 3A,B, respectively). Interestingly, in these experiments, we noticed that autophagy was not modulated in the neighboring uninfected cells, confirming a direct effect of the virus on HK2 cells. We then performed immunoblotting to detect LC3 in HCMV-infected HK-2 cells. During autophagy, the cytosolic LC3 form (LC3-I) undergoes a modifying process to a phosphatidylethanolamine-conjugated form (LC3-II), which can bind to the autophagosomal membranes (Klionsky et al., 2016). LC3-I and LC3-II are easily separated by SDS-PAGE because of their difference in electrophoretic mobility, and the expression of LC3-II correlates with the level of autophagy. To assess the autophagic flux, we used chloroquine (CQ), which neutralized the lysosomal pH and by inhibiting endogenous protein breakdown caused the accumulation of LC3-II in both autophagosomes and autolysosomes (Klionsky et al., 2016). HK-2 cells were mock- or HCMV-infected for 2 and 7 days and when indicated, treated with CQ and/or EBSS for the last 4 h of infection. In mock-infected cells, we observed an increase of LC3-II expression in EBSS-treated cells (Figure 3). Moreover, the addition of CQ led to an accumulation of LC3-II, reflecting the autophagic flux. In HCMV-infected cells treated with EBSS, a decrease in LC3-II expression was observed after 2 days of infection compared with the mock-infected EBSS-induced samples (Figure 3C). Interestingly, in HCMV-infected cells grown in complete medium, we observed a gradual slowdown in the autophagic flux, since the ratio between with and without CQ conditions is lower (1.8 and 1.1 for 2 and 7 d, respectively) compared to the one observed in the mock counterparts (3.8 and 1.2 for 2 and 7 d, respectively). At 7 d p.i. we failed to detect IEA by immunoblot, which we attributed to the fact that the percentage of infected-cells was too low to detect viral protein expression by Western blot. Nevertheless, we confirmed the infection by detection of IEA by immunofluorescence assay (data not shown). Taken together, these data indicate that HCMV inhibits the autophagy pathway of renal HK-2 cells.

### HCMV Induces the Enlargement of Renal Tubular Epithelial Cells

**Figure 4:**
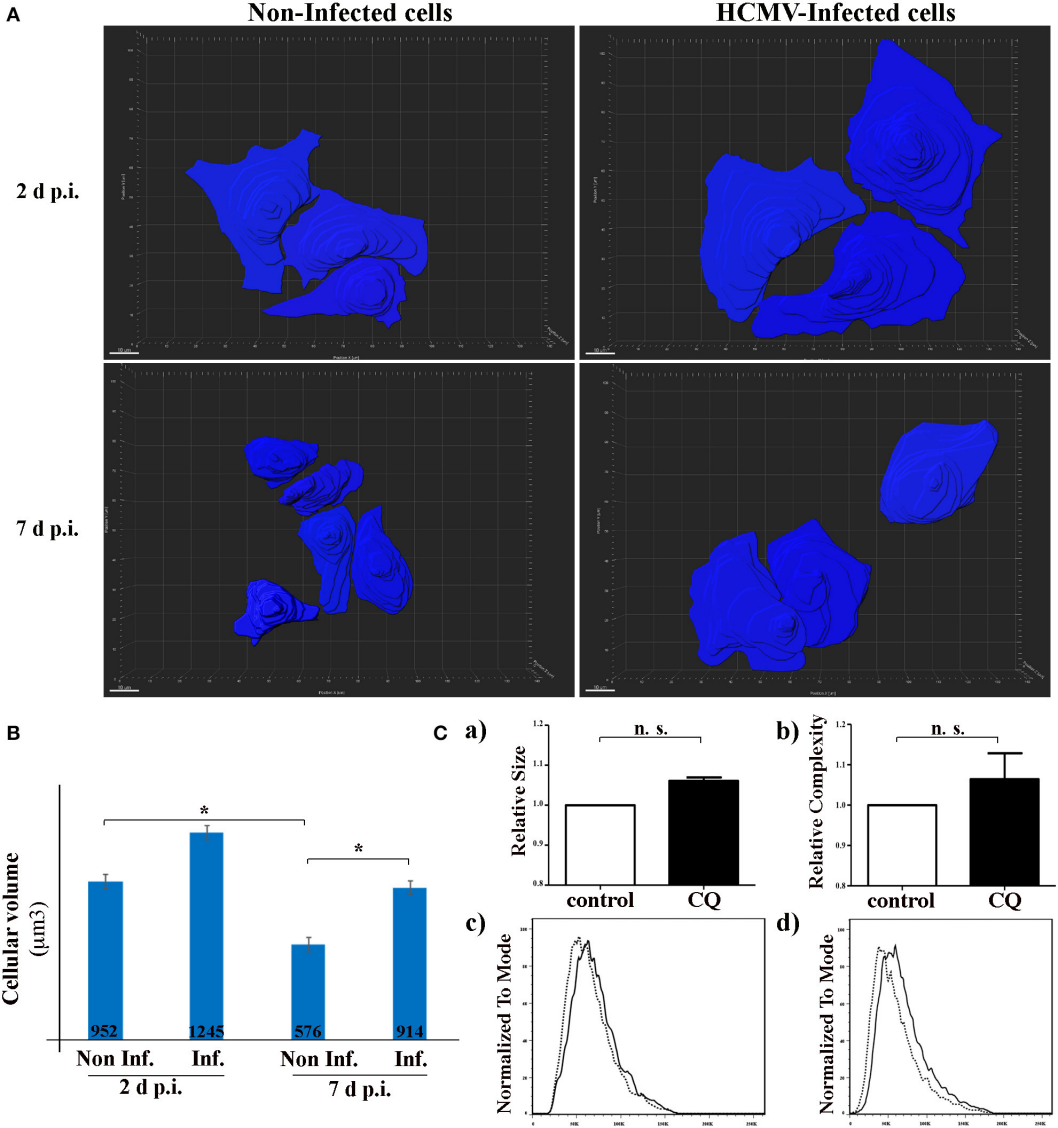
HCMV controls the size of HK-2 cells during infection. **(A)** 3D reconstruction of non-infected- and HCMV-infected HK-2 cells, 48 h and 7 d p.i., using over-saturated LC3 staining images to observe the complete cell and Imaris Software to represent cell size. Scale bar represent 10 μm **(B)** Bar graph showing quantification of the mean cell volume of non-infected and infected cells. *p < 0.05 (ANOVA, with a Tukey contrast) **(C)** Human epithelial HeLa cells were treated for 4 h with CQ or left in control condition. Bar graphs indicating (a) size or (b) cellular complexity of cells treated with CQ or untreated (control). Representative flow cytometry histograms showing the size (c) or cellular complexity (d) of HeLa cells treated with CQ or untreated (control). Images are representative of three independent experiments.

It has been recently indicated *in vitro* and *in vivo* that autophagy is a major regulator in the cell-volume regulation in kidney epithelial cells under fluid flow (Orhon et al., 2016b). They demonstrated that inhibition of autophagy leads to an enlargement of renal epithelial cell size. Since HCMV infection causes enlargement of the cell (cytomegaly), we hypothesized that inhibition of autophagy by HCMV could lead to an increase of the size of renal tubular epithelial cells. To address this point, we used SIM to acquire the z-stack images of the cells in different visual fields of the μ-slide (Figure 4). Then, the 3D reconstruction images to compare the mean cell volume of non-infected and infected HK-2 cells, 2 and 7 days p.i. (Figures 4A,B). We observed an enlargement of infected cells at both 2 and 7 days p.i. However, the difference in the cellular volume was statistically significant only at 7 d p.i. Of note, a significant decrease in the cell size was observed in non-infected cells 7 days p.i. regarding 2 days p.i., that could be explained by the polarization process which produces a greater cellular density on the plastic substratum, with a smaller individual cell volume as a consequence. Orhon and collaborators demonstrated that CQ administration to mice, which blocks the autophagic flux, resulted in increased epithelial cell size in kidney (Orhon et al., 2016b). Thus, we used FACS, a cellular population analysis technique, to study epithelial cell size in response to inhibition of autophagy by CQ. For this purpose, we decided to use human epithelial HeLa cells since (i) they are suitable for FACS and (ii) they are more widely used than the renal cell line to study cellular processes. As observed in Figure 4C, we detected an enlargement of the cell size after CQ-treatment of epithelial cells, confirming the notion that enlargement of the cells could be related to inhibition of the autophagic flux.

## Discussion

Kidney transplant (KT) is the best treatment option for patients with a end-stage chronic kidney disease increasing the duration and quality of life for patients (Wolfe et al., 1999). Worldwide, the number of transplants performed in the world is increasing every year and renal allograft is the most common one. However, post-transplant immunosuppressive drugs is inevitably associated with opportunistic viral infections, mainly by persistent viruses such as the members of the *Herpesviridae* family. Especially, HCMV, Epstein–Barr virus (EBV) and human herpesvirus type 6 (HHV-6) are of interest, since they can cause severe post-transplant complications (Moal et al., 2013). Antiviral agents are recommanded to prevent the viral multiplication and the appearance of clinical diseases, but they are unable to eradicate these viruses. In this context, HCMV infection is the most frequent complication in KT because HCMV replicates to high rates, causing both direct effects, with viral replication in the peripheral blood and organ damage and indirect effects, characterized by a most frequent kidney dysfunction and a risk of organ rejection. (De Keyzer et al., 2011; Requião-Moura et al., 2015; López-Oliva et al., 2017).

In this work, we used the established HK-2 cell line of human immortalized renal tubular epithelial cells (Ryan et al., 1994) to study host-cell interaction aspects which could contribute to elucidating HCMV-induced renal pathophysiology. HK-2 cells, like MDCK (canis renal-derived cell line) cells, start polarizing as soon as they reach confluency over the substratum, thus mimicking the morphology of the urinary tract epithelia. Although the species of the two cell lines are different, both renal tubule cell lines have been used in invaluable systems for *in vitro* analyses of various tubule segment-specific physiological and biochemical functions (Ng et al., 2003; Liu et al., 2008). We observed a significant level of infection by HCMV of HK-2 cells. But, as shown here, the percentage of HCMV-infected cells was lower when the infection was performed on a polarized monolayer of HK-2 cells. This result is in agreement with previous observations made in human intestinal epithelial Caco-2 cells which mimics enterocyte (Esclatine et al., 2000, 2001). Caco-2 cells, upon reaching confluency, spontaneously become polarized and differentiate to display typical morphogical and functional features of small intestine enterocytes (Pinto et al., 1983). In these studies, the authors demonstrated that HCMV infection of polarized Caco-2 cells is poorly efficient because it proceeds preferentially through the basolateral membrane (Esclatine et al., 2000, 2001). This phenomenon is associated with a redistribution of viral receptors to this side of the cell during polarization. In this context, we hypothesize that, when HK-2 cells are non-polarized, the receptors are equally distributed all along the cellular membrane promoting HCMV entry while they redistribute to the basolateral membrane in polarized cells, becoming inaccessible to the virus in normal culture conditions.

We observed here that infection of HK-2 cells by HCMV leads to an increase in the cell size and that HCMV infection inhibits the autophagy pathway. This can be correlated to a recent study demonstrating a role of autophagy in the regulation of the cell size (Orhon et al., 2016a). In this study, the authors showed that the sensing of fluid flow by PC induces autophagy which decreases cell volume of epithelial renal cells. In the kidney, PC plays an important role in the maintenance of renal function and structure and it has been demonstrated that the length of the PC is dynamically changed in cells as a result of various kidney diseases (Park, 2018). Thus, we studied both ciliogenesis and PC structure in infected cells but we observed that neither of them was affected by the presence of the virus. Therefore, the inhibition of autophagy and the cell enlargement observed in HCMV-infected HK2 cells seem to be PC-independent.

Nevertheless, we hypothesize a link between cell enlargement and inhibition of autophagy during HCMV infection. Indeed, using a chemical treatment approach in human epithelial cells, we observed that blocking autophagy with CQ led to an increased cellular volume of the treated cells.

The autophagy involvement in the regulation of cell size in response to amino acid concentration fluctuation and osmosensing has been observed by pioneering studies in the liver (Meijer et al., 1993; Vom Dahl et al., 2003). Moreover, several more-recent studies reported that genetic defect of autophagy genes can lead to an increase in cell size in various cells and tissues such as mouse embryo fibroblasts (Jin et al., 2015), the kidney proximal tubule (Kimura et al., 2011; Orhon et al., 2016b), the liver (Komatsu et al., 2005; Jin et al., 2015) and the Drosophila intestine cell size (Chang et al., 2013) strongly suggesting that the function of autophagy in cell volume control is evolutionarily conserved. An increase of cell volume is a frequent characteristic observed during renal failure. Thus, mechanims allowing the regulation of these morphogy changes could have a crucial role in the physiopahtology of renal failure and need to be explored.

In conclusion, our main contribution is based on the establishment of a renal tubular epithelial model to study HCMV-host interaction to understand the viral-induced physiopathological effects underlying the post-kidney allograft failure. We did a great effort to demonstrate HCMV TB40/E viral replication/propagation but systematically failed to observe this, so it is highly probable that HK-2 cells infection with TB40/E is abortive. Nevertheless, the observed HCMV-dependent autophagy inhibition and increased cell volume triggered by the presence of the virus in the cells constitute an excellent starting point to further explore the role of both mechanisms, in renal cells dysfunction. Indeed, for autophagy, there is considerable, but still unrealized, potential for translating preclinical findings on autophagy modulation into a therapeutic benefit for different patient populations (Rubinsztein et al., 2012).

## Data Availability Statement

All datasets generated for this study are included in the article/supplementary material.

## Author Contributions

AL, EH, MT, CT, JR, CG, ML, and LD performed the experiments. AE and LD prepared the manuscript. PC and MC were mentors of the work and contributed to manuscript edition. All authors contributed to the article and approved the submitted version.

## Conflict of Interest

The authors declare that the research was conducted in the absence of any commercial or financial relationships that could be construed as a potential conflict of interest.
